# Trajectory clustering of immune cells and its association with clinical outcomes after aneurysmal subarachnoid hemorrhage

**DOI:** 10.3389/fneur.2024.1491189

**Published:** 2024-11-05

**Authors:** So Young Won, Museong Kim, Han-Gil Jeong, Bosco Seong Kyu Yang, Huimahn Alex Choi, Dong-Wan Kang, Yong Soo Kim, Young Deok Kim, Si Un Lee, Seung Pil Ban, Jae Seung Bang, Moon-ku Han, O-Ki Kwon, Chang Wan Oh

**Affiliations:** ^1^Division of Neurocritical Care, Department of Neurosurgery and Neurology, Seoul National University Bundang Hospital, Seoul National University College of Medicine, Seongnam-si, Republic of Korea; ^2^Department of Neurosurgery, McGovern Medical School, The University of Texas Health Science Center at Houston, Houston, TX, United States; ^3^Department of Neurosurgery, Seoul National University Bundang Hospital, Seoul National University College of Medicine, Seongnam-si, Republic of Korea

**Keywords:** subarachnoid hemorrhage, neutrophil, monocyte, lymphocyte, neuroinflammation, cluster analysis

## Abstract

**Background and purpose:**

The immune response following aneurysmal subarachnoid hemorrhage (aSAH) can exacerbate secondary brain injury and impact clinical outcomes. As the immune response after aSAH is a dynamic process, we aim to track and characterize immune cell trajectories over time to identify patterns associated with various clinical outcomes.

**Methods:**

In this retrospective single-center study of patients with aSAH, we analyzed immune cell count trajectories, including neutrophil, monocyte, and lymphocyte counts, collected from day 1 to day 14. These trajectories were classified into four distinct clusters utilizing the k-means longitudinal clustering method. A comprehensive multivariable analysis was performed to explore the associations of these immune cell clusters with various clinical outcomes. These outcomes included a Modified Rankin Scale score (mRS) of 3 to 6, indicative of poor functional outcomes, along with complications including shunt dependency, vasospasm, and secondary cerebral infarction.

**Results:**

In this study, 304 patients with aSAH were analyzed. The trajectories of immune cell counts, including neutrophils, monocytes, and lymphocytes, were successfully categorized into four distinct clusters for each immune cell type. Within neutrophil clusters, both persistent neutrophilia and progressive neutrophilia were associated with poor functional outcomes, shunt dependency, and vasospasm, with resolving neutrophilia showing a lesser degree of these associations. Within monocyte clusters, early monocytosis was associated with vasospasm, whereas delayed monocytosis was associated with shunt dependency. Within lymphocyte clusters, both early transient lymphopenia and early prolonged lymphopenia were associated with poor functional outcomes.

**Conclusion:**

Our study demonstrates that distinct immune cell trajectories post-aSAH, identified through unsupervised clustering, are significantly associated with specific clinical outcomes. Understanding these dynamic immune responses may provide key insights with potential for future therapeutic strategies.

## Introduction

Aneurysmal subarachnoid hemorrhage (aSAH), a form of stroke characterized by bleeding into the subarachnoid space, can be complicated by extension into the ventricles or brain parenchyma, and remains a significant clinical challenge (1). Despite advancements in acute management, aSAH continues to result in high morbidity and mortality rates (2–4). One of the critical unmet needs in aSAH treatment is the development of interventions aimed at mitigating brain injury to improve patient outcomes. The complexity of aSAH pathophysiology, which involves intricate interactions between vascular, neuronal, and immune systems, underscores the necessity for a sophisticated approach to bridge the gaps in care (5, 6).

The aftermath of aSAH triggers a complex immune response, initiated by the presence of toxic blood products and early brain injury (7). The degradation of red blood cells within the subarachnoid space releases substances such as methemoglobin and free heme, which are known to provoke a potent inflammatory reaction (8, 9). In addition, the release of damage-associated molecular patterns and activation of the sympathetic nervous system stimulate the bone marrow and spleen, resulting in the efflux of immune cells such as neutrophils and monocytes (10). These peripheral immune cells can infiltrate into the brain parenchyma and exacerbate the inflammatory milieu (8, 11). Recent studies have underscored the importance of this peripheral immune activation, linking it to key pathophysiological processes such as delayed cerebral ischemia and long-term neurological deficits (12, 13).

Variability in the peripheral immune response after aSAH is influenced by factors such as the extent of cerebral injury, host characteristics, surgical interventions, complications such as infection (10). Hence, peripheral immune responses in aSAH patients exhibit a dynamic nature, varying significantly both across individuals and over time (14). This variability presents a challenge: understanding and categorizing the peripheral immune response could be key to improving patient management (15, 16). Recognizing this, our study aimed to investigate whether the trajectories of immune cell counts post-aSAH could be clustered in an unsupervised manner and whether these clusters have specific associations with various clinical outcomes.

## Methods

### Patient selection

This retrospective study analyzed patient data collected from April 2013 to May 2022 at the Seoul National University Bundang Hospital’s Neurocritical Care Unit. The inclusion criteria were as follows: (1) patients with aSAH; (2) admission to an intensive care unit within 24 h of initial symptom onset; (3) receipt of aneurysm repair treatment; (4) availability of white blood cell counts for at least 4 days including day 1 during the first 14 days after aSAH; (5) a premorbid modified Rankin Scale (mRS) score of 0 to 2. Institutional review board committee approval was obtained; however, the need for informed consent was permitted to be waived, given the retrospective nature of the study.

### Clinical and laboratory data collection and definition

We collected baseline demographics, medical history (including premorbid mRS, hypertension, diabetes mellitus), clinical characteristics, descriptive radiologic imaging findings, and clinical outcomes from electronic health records. The clinical severity of patients was assessed using Hunt & Hess classification and World Federation of Neurosurgical Societies grade. The first brain computed tomography (CT) scan within 24 h after aSAH was used to measure the modified Fisher scale, and to assess the presence of intracerebral hemorrhage, symptomatic hydrocephalus, global cerebral edema, and the subarachnoid hemorrhage early brain edema score. Using digital subtraction angiography (DSA), we evaluated the number (single or multiple), location, and size of aneurysms. The decision to undergo surgical clipping or endovascular coiling of the ruptured aneurysm was made at the physician’s discretion. Treatment information included the time from onset to aneurysm treatment, treatment modality (surgical or endovascular), external ventricular drainage, and lumbar drainage. Functional outcome at 6 months after aSAH was evaluated using mRS, documented either by telephone interview or in outpatient records. In our study, white blood cell (WBC) counts were collected following a specific time-frame protocol to ensure consistency and precision. The initial count, termed ‘day 0’, was taken within the first 12 h post-symptom onset. Subsequent counts were scheduled to approximate 24-h intervals: ‘day 1’ counts were recorded between 12 to 36 h, ‘day 2’ counts between 36 to 60 h, and so forth. In instances where multiple WBC counts were available for a given time period, the value closest to the 24, 48, 72-h marks, etc., was selected. This method was employed to align the counts as closely as possible with the intended time points post-symptom onset. These WBC values, extracted from the electronic health records, were part of routine patient care and management.

### Outcomes

Unfavorable functional outcome was defined using mRS score of 3 to 6 at 6 months. Transcranial *Doppler* (TCD) vasospasm is defined as mean flow velocity over 120 m/s or *Lindegaard* ratio over 3. Angiographic vasospasm is defined as present when the diameter of the distal ICA, A1 and M1 segment are reduced by more than 50%, as evaluated by CT angiography or DSA (17, 18). Intra-arterial spasmolysis, mainly the infusion of nimodipine, was performed in patients showing angiographic evidence of vasospasm in a territory correlating with their neurologic deficit or in those exhibiting substantially elevated mean flow velocities in TCD studies. It was predominantly injected on the A1 segment of the anterior cerebral artery or the M1 segment of the middle cerebral artery. Shunt dependency was defined when ventriculoperitoneal shunt is conducted within 6 months after ictus. Secondary cerebral infarction is diagnosed when it is not observed in the postprocedural CT scan performed after aneurysm occlusion but is present in the last cerebral imaging (either CT or magnetic resonance imaging) conducted before discharge from acute care.

### Statistical analysis

Baseline characteristics of the included patients were compared and summarized using *χ*^2^ tests for categorical variables and Analysis of Variance (ANOVA) for continuous variables. The trajectory of immune cells was clustered into four groups using k-means longitudinal clustering method, an unsupervised clustering method for clustering time series data where time points are treated as variables in classic k-means clustering (19). Bivariate analysis was conducted to compare each cluster, followed by multivariable logistic regression, which was adjusted for covariates that are clinically relevant or had a *p*-value of less than 0.2 in the bivariate analysis. The full list of adjusted covariates for each model is provided in the results of the multivariable analyses. Statistical significance was set at a *p*-value of 0.05. All the statistical analyses were performed using R statistical software (version 4.3.2), and the “kml” package was specifically used for k-means longitudinal clustering.

## Results

### Patient characteristics

**Figure 1:**
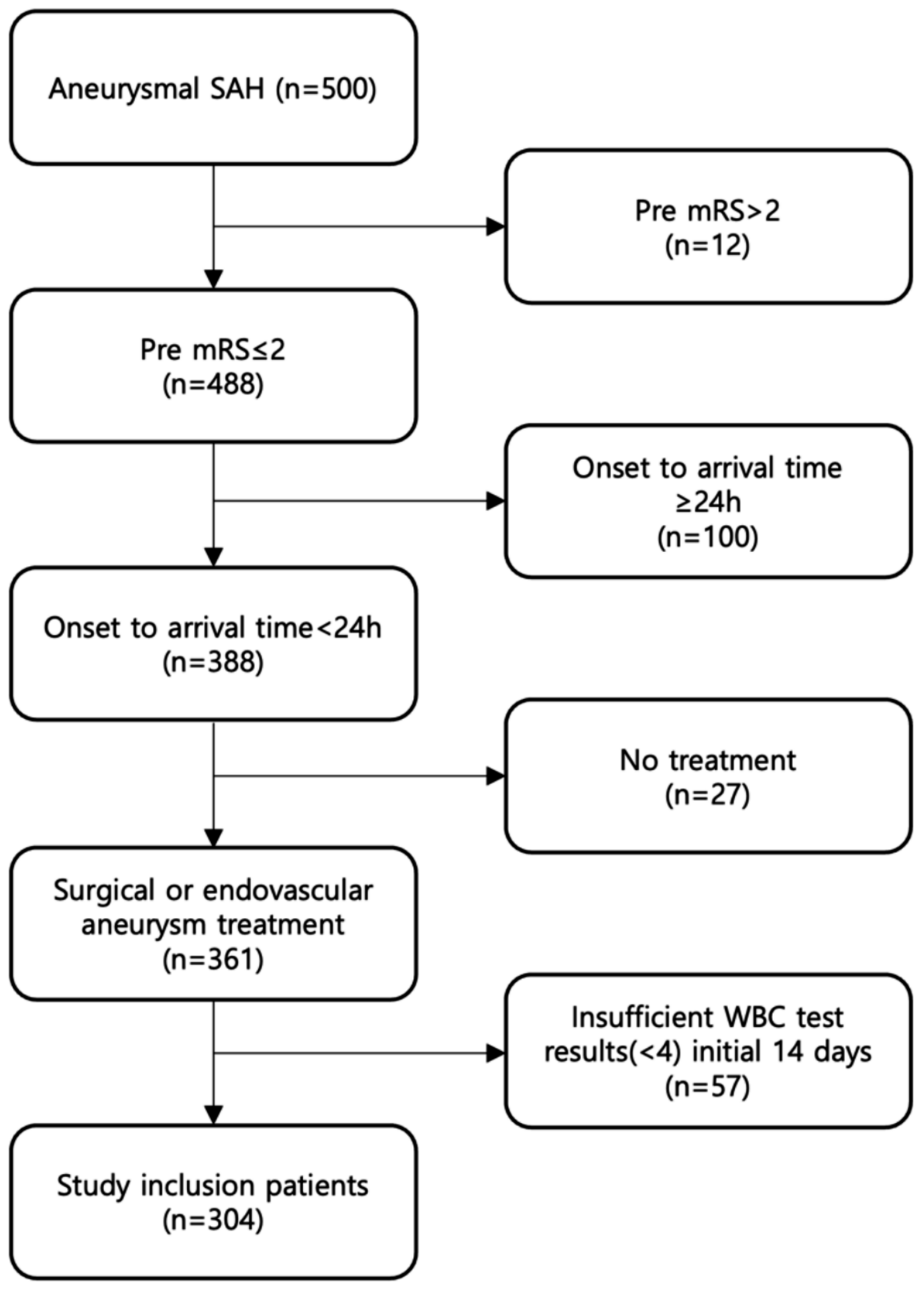
Eligible patients in the study. We identified 500 patients diagnosed with aSAH between April 2013 and May 2022, and included total 304 patients who met the inclusion criteria.

Among 500 patients aSAH in our hospital, 304 patients (99 men, 205 women; mean age, 57 ± 13 years) meeting the inclusion criteria were finally analyzed (Figure 1). The baseline characteristics and clinical outcomes of the included patients are summarized in Table 1.

**Table 1:** Patient baseline characteristics and clinical features.

Characteristics	Overall (*n* = 304)
Age, years	57.0 ± 13.0
Sex	
Female	205 (67.4)
Male	99 (32.6)
Premorbid mRS	
0	294 (96.7)
1	7 (2.3)
2	3 (1.0)
History of hypertension	128 (42.1)
History of diabetes	27 (8.9)
Hunt & Hess classification	
1	50 (16.4)
2	85 (28.0)
3	85 (28.0)
4	59 (19.4)
5	25 (8.2)
WFNS grade	
1	131 (43.1)
2	53 (17.4)
3	15 (4.9)
4	61 (21.1)
5	41 (13.5)
Modified Fisher scale	
1	60 (19.7)
2	19 (6.2)
3	114 (37.5)
4	111 (36.5)
Intracerebral hemorrhage	63 (20.7)
Global cerebral edema	108 (35.8)
Subarachnoid hemorrhage early brain edema score	
0	18 (6.0)
1	18 (6.0)
2	104 (34.4)
3	58 (19.2)
4	104 (34.4)
Aneurysm location	
ACA	14 (4.6)
ACoA	103 (33.9)
ICA	29 (9.5)
MCA	69 (22.7)
PCoA	61 (20.1)
PCA	3 (1.0)
BA	8 (2.6)
VA/Cerebellar	17 (5.6)
Aneurysm circulation	
Anterior	276 (90.8)
Posterior	28 (9.2)
Onset to arrival time, h	1.6 [0.7–4.3]
Onset to aneurysm treatment time, h	7.0 [4.8–11.3]
Aneurysm treatment modality	
Surgical	136 (44.7)
Endovascular	168 (55.3)
External ventricular drainage	100 (32.9)
Lumbar drainage	142 (46.7)
TCD vasospasm	165 (59.4)
Angiographic vasospasm	100 (34.8)
Intra-arterial spasmolysis	65 (21.4)
Secondary infarction	56 (18.4)
Shunt dependency	58 (19.1)
mRS score 3–6 at 6 month	102 (33.4)

Values are *n* (%), mean ± standard deviation, or median [interquartile range]; mRS, modified Rankin Score; WFNS, World Federal Neurosurgical Society; ACA, anterior cerebral artery; AcoA, anterior communicating artery; ICA, internal carotid artery; MCA, middle cerebral artery; PCoA, posterior communicating artery; PCA, posterior cerebral artery; BA, basilar artery; VA, vertebral artery; TCD, transcranial Doppler. One patient’s mRS score at 6 months was lost due to a loss of follow-up after discharge.

### Neutrophil clusters and outcomes

**Figure 2:**
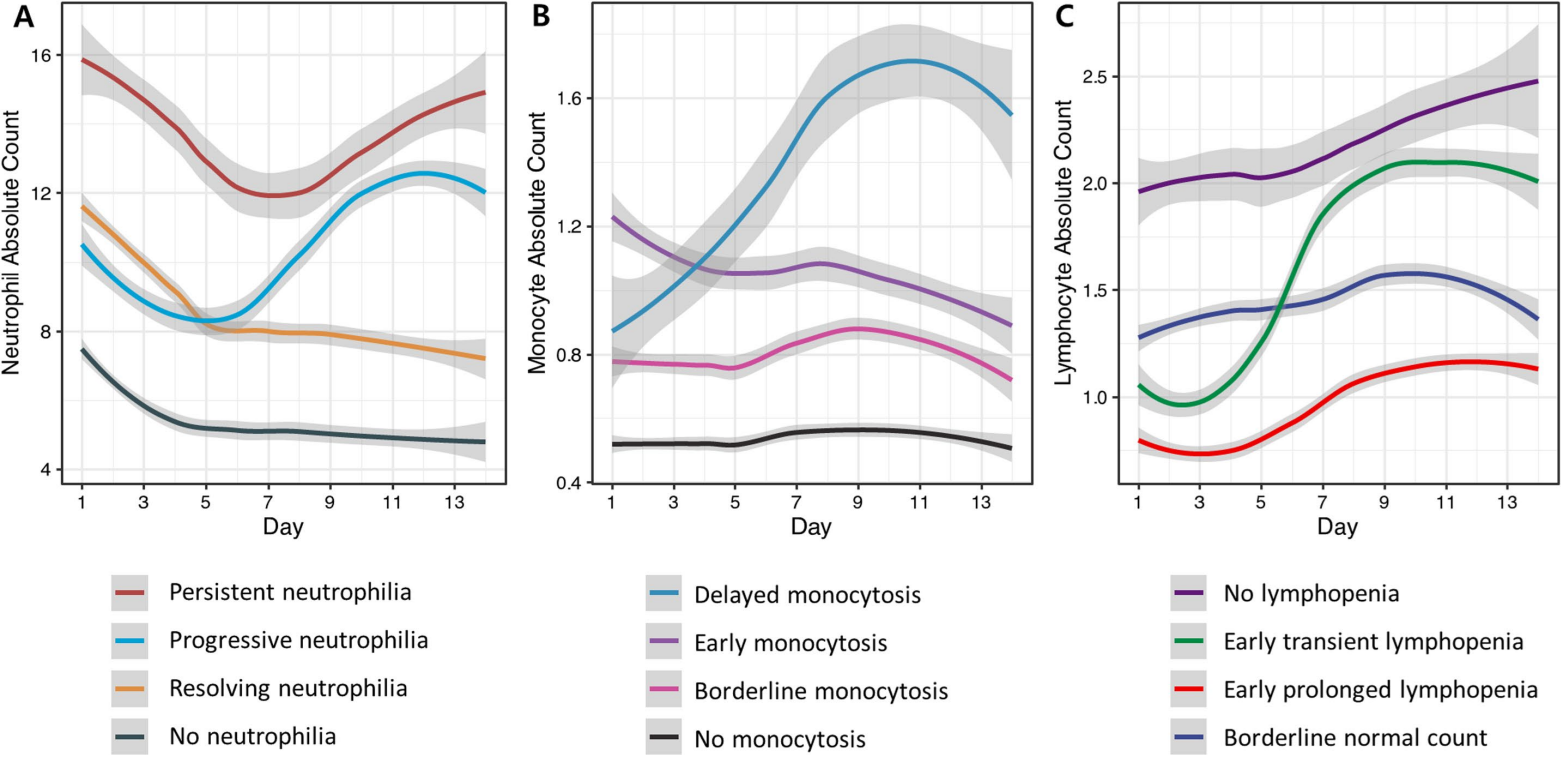
Clustering of blood neutrophil, monocyte, and lymphocyte trajectories from day 1 to day 14 after aSAH. The cell counts are presented in units of 1,000 per microliter. Each line in the graph represents the average trend for each trajectory cluster, estimated using locally estimated scatterplot smoothing. The shaded areas around each line signify the 95% confidence intervals for these estimations, providing insight into the variability and reliability of the trends. **(A)** Neutrophil trajectories were classified into persistent elevation (red), progressive elevation (light blue), early elevation followed by gradual resolution (yellow), and no elevation (grey). **(B)** Monocyte trajectories were classified into delayed elevation (blue), early significant elevation (purple), borderline elevation (pink), and no elevation (black). **(C)** Lymphocyte trajectories were classified as normal lymphocyte count (dark purple), early but transient decrease (green), early and sustained decrease (red), and borderline elevation (dark blue).

**Figure 3:**
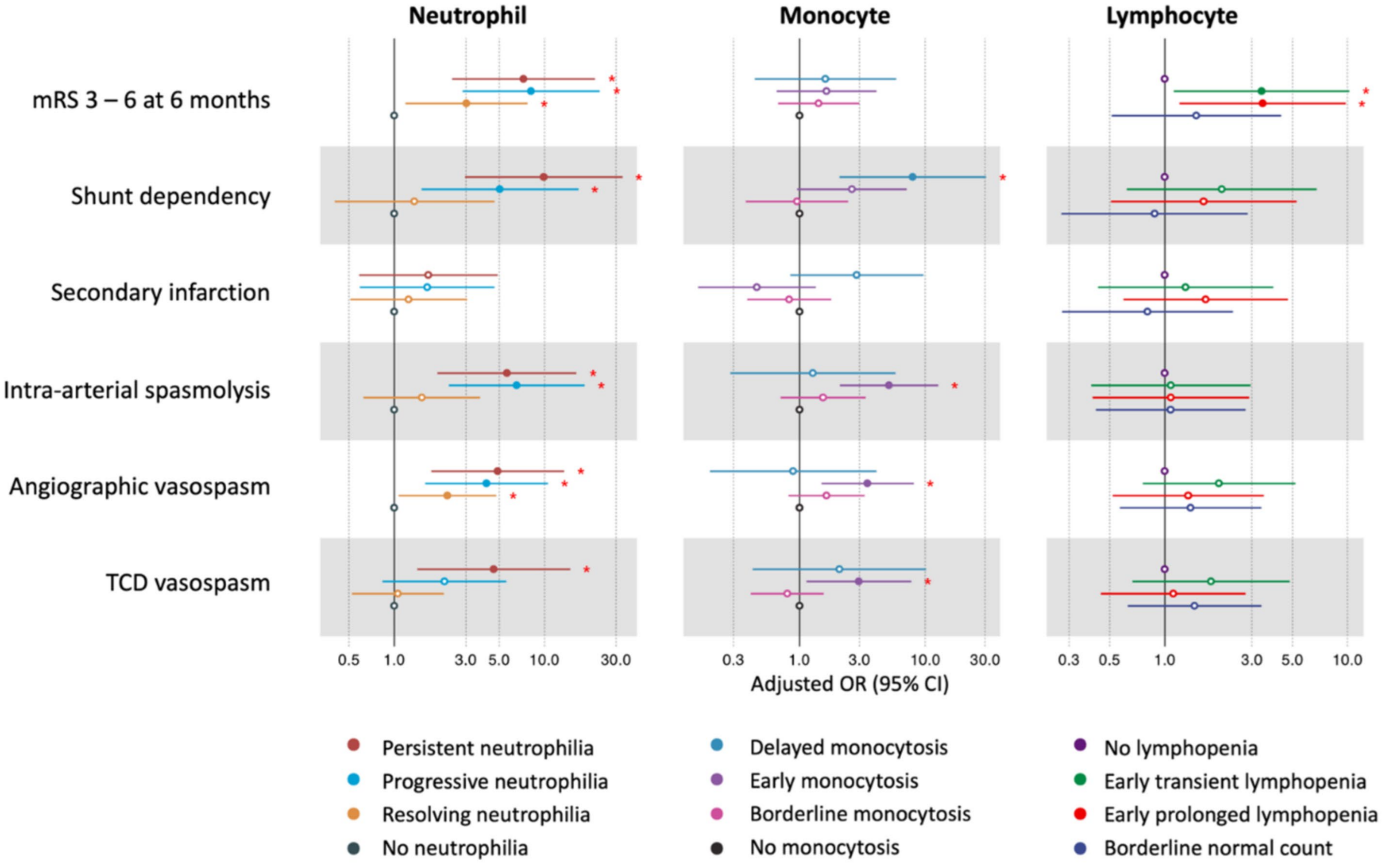
The association between neutrophil, monocyte, lymphocyte clusters, and outcomes after aSAH. The red asterisks (*) indicate statistically significant associations at the *p* < 0.05 level. Neutrophil clusters were adjusted for age, sex, premorbid mRS, Hunt & Hess classification, WFNS grade, modified Fisher scale, intracerebral hemorrhage, aneurysm treatment modality, EVD, and lumbar drainage. Monocyte clusters were adjusted for age, sex, history of diabetes, Hunt & Hess classification, WFNS grade, modified Fisher scale, intracerebral hemorrhage, aneurysm treatment modality, and EVD. Lymphocyte clusters were adjusted for age, sex, history of hypertension, Hunt & Hess classification, WFNS grade, intracerebral hemorrhage, global cerebral edema, subarachnoid hemorrhage early brain edema score, and aneurysm treatment modality. mRS, modified Rankin Scale; TCD, transcranial *Doppler*; OR, odds ratio; CI, confidence interval; WFNS, World Federation of Neurosurgical Societies; EVD, external ventricular drainage.

Neutrophil trajectories were successfully clustered into four distinct groups: ‘no neutrophilia’ (35.8%), ‘resolving neutrophilia’ (31.6%), ‘progressive neutrophilia’ (19.7%), and ‘persistent neutrophilia’ (12.8%) (Figure 2A). The comparison of patient characteristics between each cluster is presented in Table 2. In the multivariable analysis, the following associations were observed (Figure 3 and Supplementary Table S3): Unfavorable functional outcomes at 6 months were associated with progressive neutrophilia (adjusted odd ratio [aOR] 8.14, 95% confidence interval [CI] 2.84–23.32), persistent neutrophilia (aOR 7.24, 95% CI 2.42–21.65), and resolving neutrophilia (aOR 3.02, 95% CI 1.19–7.71). Shunt dependency was associated with persistent neutrophilia (aOR 9.89, 95% CI 2.96–33.07) and progressive neutrophilia (aOR 5.05, 95% CI 1.51–16.84). Intra-arterial spasmolysis was associated with progressive neutrophilia (aOR 6.53, 95% CI 2.30–18.51) and persistent neutrophilia (aOR 5.62, 95% CI 1.94–16.30). Angiographic vasospasm was associated with persistent neutrophilia (aOR 4.88, 95% CI 1.76–13.52), progressive neutrophilia (aOR 4.11, 95% CI 1.60–10.55), and resolving neutrophilia (aOR 2.25, 95% CI 1.60–4.78). TCD vasospasm was associated with persistent neutrophilia (aOR 4.58, 95% CI 1.41–14.81). No association was found between secondary infarction and any neutrophil cluster.

**Table 2:** Comparison of patient characteristics and outcomes across neutrophil cluster.

Characteristic	No neutrophilia (*n* = 109, 35.8%)	Resolving neutrophilia (*n* = 96, 31.6%)	Progressive neutrophilia (*n* = 60, 19.7%)	Persistent neutrophilia (*n* = 39, 12.8%)	*p*-value
Age, years	56.8 ± 10.7	56.6 ± 13.4	59.1 ± 15.5	55.7 ± 14.1	0.56
Sex					0.21
Female	81 (74.3)	64 (66.7)	37 (61.7)	23 (59.0)	
Male	28 (25.7)	32 (33.3)	23 (38.3)	16 (41.0)	
Premorbid mRS					0.16
0	107 (98.2)	95 (99.0)	55 (91.7)	37 (94.9)	
1	2 (1.8)	1 (1.0)	3 (5.0)	1 (2.6)	
2	0 (0.0)	0 (0.0)	2 (3.3)	1 (2.6)	
History of hypertension	50 (45.9)	39 (40.6)	26 (43.3)	13 (33.3)	0.58
History of diabetes	9 (8.3)	7 (7.3)	4 (6.7)	7 (17.9)	0.20
Hunt & Hess classification					<0.01
1	29 (26.6)	12 (12.5)	3 (5.0)	6 (15.4)	
2	45 (41.3)	26 (27.1)	12 (20.0)	2 (5.1)	
3	27 (24.8)	30 (31.2)	13 (21.7)	15 (38.5)	
4	7 (6.4)	20 (20.8)	20 (33.3)	12 (30.8)	
5	1 (0.9)	8 (8.3)	12 (20.0)	4 (10.3)	
WFNS grade					<0.01
1	74 (67.9)	35 (36.5)	12 (20.0)	10 (25.6)	
2	18 (16.5)	19 (19.8)	6 (10.0)	10 (25.6)	
3	4 (3.7)	2 (2.1)	5 (8.3)	4 (10.3)	
4	10 (9.2)	28 (29.2)	19 (31.7)	7 (17.9)	
5	3 (2.8)	12 (12.5)	18 (30.0)	8 (20.5)	
Modified Fisher scale					0.01
1	29 (26.6)	22 (22.9)	4 (6.7)	5 (12.8)	
2	7 (6.4)	7 (7.3)	4 (6.7)	1 (2.6)	
3	46 (42.2)	35 (36.5)	20 (33.3)	13 (33.3)	
4	27 (24.8)	32 (33.3)	32 (53.3)	20 (51.3)	
Intracerebral hemorrhage	13 (11.9)	18 (18.8)	19 (31.7)	13 (33.3)	<0.01
Global cerebral edema	32 (29.4)	33 (34.7)	26 (44.1)	17 (43.6)	0.19
Subarachnoid hemorrhage early brain edema score					0.30
0	8 (7.3)	4 (4.2)	5 (8.5)	1 (2.6)	
1	7 (6.4)	4 (4.2)	4 (6.8)	3 (7.7)	
2	46 (42.2)	35 (36.8)	15 (25.4)	8 (20.5)	
3	17 (15.6)	20 (21.1)	10 (16.9)	11 (28.2)	
4	31 (28.4)	32 (33.7)	25 (42.4)	16 (41.0)	
Aneurysm location					0.78
ACA	6 (5.5)	3 (3.1)	2 (3.3)	3 (7.7)	
ACoA	37 (33.9)	36 (37.5)	19 (31.7)	11 (28.2)	
ICA	11 (10.1)	6 (6.2)	7 (11.7)	5 (12.8)	
MCA	19 (17.4)	24 (25.0)	17 (28.3)	9 (23.1)	
PCoA	26 (23.9)	17 (17.7)	10 (16.7)	8 (20.5)	
PCA	3 (2.8)	0 (0.0)	0 (0.0)	0 (0.0)	
VA/Cerebellar	4 (3.7)	8 (8.3)	3 (5.0)	2 (5.1)	
BA	3 (2.8)	2 (2.1)	2 (3.3)	1 (2.6)	
Aneurysm circulation					0.95
Anterior	99 (90.8)	86 (89.6)	55 (91.7)	36 (92.3)	
Posterior	10 (9.2)	10 (10.4)	5 (8.3)	3 (7.7)	
Onset to arrival time, h	2.0 [0.8–5.2]	1.9 [0.7–4.4]	1.3 [0.6–3.3]	1.5 [0.7–2.2]	0.50
Onset to treatment time, h	8.4 [5.3–12.5]	7.0 [5.0–10.6]	6.0 [4.6–96.0]	7.4 [4.8–10.1]	0.75
Aneurysm treatment modality					<0.01
Surgical	23 (21.1)	2 (2.1)	39 (65.0)	22 (56.4)	
Endovascular	86 (78.9)	94 (97.9)	21 (35.0)	17 (43.6)	
External ventricular drainage	14 (12.8)	33 (34.4)	30 (50.0)	23 (59.0)	<0.01
Lumbar drainage	62 (56.9)	44 (45.8)	20 (33.3)	16 (41.0)	0.03
TCD vasospasm	48 (47.5)	53 (59.6)	37 (67.3)	27 (81.8)	<0.01
Angiographic vasospasm	20 (18.5)	34 (37.8)	25 (47.2)	21 (58.3)	<0.01
Intra-arterial spasmolysis	12 (11.0)	17 (17.7)	20 (33.3)	16 (41.0)	<0.01
Secondary infarction	13 (11.9)	17 (17.7)	15 (25.0)	11 (28.2)	0.06
Shunt dependency	6 (5.5)	10 (10.4)	21 (35.0)	21 (35.0)	<0.01
mRS score 3–6 at 6 month	11 (10.1)	31 (32.3)	38 (64.4)	22 (56.4)	<0.01

Values are *n* (%), mean ± standard deviation, or median [interquartile range]. mRS, modified Rankin Score; WFNS, World Federal Neurosurgical Society; ACA, anterior cerebral artery; AcoA, anterior communicating artery; ICA, internal carotid artery; MCA, middle cerebral artery; PCoA, posterior communicating artery; PCA, posterior cerebral artery; BA, Basilar artery; VA, Vertebral artery; TCD, transcranial Doppler.

### Monocyte clusters and outcomes

Monocyte trajectories in the study were successfully categorized into four distinct groups: ‘no monocytosis’ (45.7%),’ ‘borderline monocytosis’ (31.9%), ‘early monocytosis’ (16.1%), and ‘delayed monocytosis’ (6.3%) (Figure 2B). Details comparing patient characteristics across these clusters are provided in Supplementary Table S1. In the multivariable analysis, the following associations were observed (Figure 3 and Supplementary Table S3): The early monocytosis group showed significant associations with intra-arterial spasmolysis (aOR 5.13, 95% CI 2.09–12.62), angiographic vasospasm (aOR 3.48, 95% CI 1.50–8.07), and TCD vasospasm (aOR 2.97, 95% CI 1.14–7.75). The delayed monocytosis group was associated with shunt dependency (aOR 7.94, 95% CI 2.08–30.29). However, no associations were found between any of the monocyte clusters and functional outcome or the occurrence of secondary infarction.

### Lymphocyte clusters and outcomes

Lymphocyte trajectories were successfully clustered into four distinct groups: ‘early prolonged lymphopenia’ (17.8%), ‘early transient lymphopenia’ (32.9%), ‘borderline normal lymphocyte count’ (20.1%), and ‘no lymphopenia’ (29.3%) (Figure 2C). The comparison of patient characteristics for each cluster is presented in Supplementary Table S2. In the multivariable analysis, the following associations were observed (Figure 3 and Supplementary Table S3): Patients in the early prolonged lymphopenia group (aOR 3.43, 95% CI 1.20–9.80) and the early transient lymphopenia group (aOR 3.39, 95% CI 1.12–10.22) were associated with unfavorable functional outcomes at 6 months. However, there were no associations observed between any lymphocyte clusters and shunt dependency, vasospasm, or secondary infarction.

## Discussion

In this study, we successfully classified immune cell trajectories post-aSAH into four distinct clusters, each demonstrating specific associations with various clinical outcomes. The presence of progressive or persistent neutrophilia was notably associated with poorer functional outcomes at 6 months, as well as with increased instances of shunt dependency and vasospasm. Early monocytosis was linked to vasospasm, while delayed monocytosis tended to be associated with shunt dependency. Additionally, early lymphocytopenia was consistently associated with unfavorable functional outcomes at 6 months, highlighting the impact of early immune responses on patient prognosis.

Several studies have reported a correlation between the severity of neutrophilia in the early stages following aSAH and unfavorable clinical outcome, which was particularly evident in the persistent neutrophilia group in our study (15, 20, 21). A recent study categorized the WBC trajectories of SAH patients into two clusters over the first four days, where an elevated longitudinal WBC trajectory increased the risk of in-hospital mortality (22). However, a noteworthy finding in our study is that the subsequent trajectory of neutrophil count changes is also crucial, specifically distinguishing between progressive neutrophilia and resolving neutrophilia in relation to aSAH-related complications and functional outcome. Another interesting finding of our study is that the clustering delineates progressive neutrophilia as a distinct group, with its impact on various outcomes being as significant as that of persistent neutrophilia. The reasons for this delayed response are not evident in our data, but complications such as infection may have contributed to this. The increase in neutrophil count in the progressive neutrophilia group aligns with the peak incidence period of vasospasm, typically occurring around days 6–8, suggesting a potential association between progressive neutrophilia and vasospasm. The correlation between elevated neutrophil counts and the development of vasospasm in aSAH patients is well-established. Studies have shown that neutrophils may contribute to vasospasm through various mechanisms, including the release of inflammatory mediators. Moreover, neutrophil-based biomarkers, such as the neutrophil-to-albumin ratio, have demonstrated potential as predictive tools for vasospasm (23–26). In the pathogenesis of post-hemorrhagic hydrocephalus, Toll-like receptor 4-regulated cytokines and immune cells play a crucial role, where inappropriately activated neutrophils during the acute phase may potentially inflict damage to the cerebrospinal fluid (CSF) homeostatic pathways (27, 28).

In terms of monocytes, an interesting finding of our study was that, while previous studies have highlighted the association between elevated monocyte counts and adverse outcomes, our results indicate that the timing of monocytosis may be critical. The observed link between early monocytosis and vasospasm supports the notion that monocytes contribute to the inflammatory response leading to cerebral vasospasm. Conversely, the association between delayed monocytosis and shunt dependency suggests a potential role for monocytes in the postoperative healing process and the development of hydrocephalus. The correlation between elevated classical monocytes, constituting 80–90% of total monocyte population, and vasospasm was demonstrated in other studies (16, 29). The classical monocyte is an inflammatory immune cell that produces cytokines, myeloperoxidase, and superoxide, and appears to contribute to the development of vasospasm (30, 31). Monocytes also release monocyte chemoattractant protein-1, activating arterial vasoconstriction and contributing to vasospasm, thus confirming our findings regarding the relationship between monocytosis and vasospasm (32). On the contrary, a delayed increase in monocyte levels could indicate a shift towards prolonged pathological inflammation. This chronic inflammatory response is likely to lead to the formation of scar tissue and blockage of CSF drainage routes, such as the glymphatic system within brain parenchyma and meningeal lymphatics. Consequently, it could impair CSF reabsorption by promoting microglial activation and scar formation (28, 29, 33).

In the lymphocyte cluster, patients with lymphocytopenia for the first few days showed an unfavorable functional outcome at 6 months. Since lymphocytopenia renders patients susceptible to infections, including pneumonia, urinary tract infections, and colitis, it prolongs the duration of mechanical ventilation and the length of stay in the intensive care unit, leading to poor functional outcomes at 6 months (34–38). After aSAH, lymphocytopenia is induced by the activation of regulatory T-cell and predominance of type 2 helper T-cell’s anti-inflammatory activities (39). Significant decrease of CD4+ T-cells, CD8+ T-cells and natural killer cells in symptomatic vasospasm patients was demonstrated in other studies, supporting our findings (35). Therefore, it is advised to consider aSAH patients as immunocompromised and pay attention to any clinical signs of infections.

In our study, we have advantages by utilizing K-means longitudinal clustering to perform clustering in an unsupervised manner, thereby eliminating the risk of subjectively categorizing patterns of immune cells (40, 41). This method facilitated easy visualization, allowing us to successfully cluster four intuitively named temporal features without an imbalance where one cluster occupies more than 50%. This clustering was based solely on the patterns of immune cells, without any intervention from other preliminary clinical information, yet it showed relevance to clinical outcomes, including the functional outcome at 6 months. To our knowledge, this approach has not been implemented previously for peripheral immune responses following aSAH. As described above, observing the trajectories of neutrophils and monocytes provides additional insights compared to simply looking at their quantities at a chosen time point. The clustering determined by the algorithm does not allow for a consistent cutoff value to be set in each clinical setting, and setting such specific values based on data from a single center could result in more subjective values.

The identification of immune cell trajectories as potential biomarkers holds significant clinical implications. By monitoring changes in immune cell populations over time, clinicians may be able to identify patients at high risk for developing complications and initiate earlier interventions. Furthermore, these biomarkers could facilitate the development of targeted immunomodulatory therapies aimed at mitigating the harmful effects of inflammation in aSAH. Prospective studies are warranted to validate these findings and explore the clinical utility of immune cell monitoring in the management of aSAH patients.

This study, however, has several limitations. Firstly, its retrospective nature may lead to potential biases in data interpretation and limit the ability to establish causal relationships. Secondly, being a single-center study evaluated only Asian patients, the findings might not fully represent the broader population and ethnicities. These limitations warrant external validation of our conclusions in different cohorts. Thirdly, the study did not include a comprehensive workup for external factors such as infections or medications, including hormones, immunosuppressants, or anti-inflammatory agents used prior to admission, all of which can significantly influence inflammatory responses. Lastly, in k-means clustering, one challenge is determining the optimal number of clusters (k), as an inappropriate choice can lead to suboptimal clustering. Additionally, k-means assumes clusters are spherical, which may not suit complex patterns and can be further distorted by outliers, leading to misclassification.

## Conclusion

This study successfully demonstrated that peripheral immune cell (neutrophil, monocyte and lymphocyte) trajectories in patients with aSAH can be classified into four clinically meaningful clusters in an unsupervised manner. Notably, specific associations were observed between each immune cell cluster and various clinical outcomes post-aSAH. Understanding of the dynamic patterns of immune responses in aSAH may potentially uncover insights for more tailored and effective treatment strategies in the future.

## Data Availability

The original contributions presented in the study are included in the article/Supplementary material, further inquiries can be directed to the corresponding author.
