# Supplementary material for: Trajectory clustering of immune cells and its association with clinical outcomes after aneurysmal subarachnoid hemorrhage
Source: Front Neurol. 2024 Nov 5;15:1491189. doi: 10.3389/fneur.2024.1491189 (PMC11573781; doi:10.3389/fneur.2024.1491189)
Supplement: Supplementary file 1 [file Table_1.DOCX]

**Supplemental Table 1. Comparison of patient characteristics and outcomes across monocyte cluster**

|  | No  monocytosis (n=139,  45.7%) | Borderline monocytosis  (n= 97,  31.9%) | Early  monocytosis (n=49,  16.1%) | Delayed  monocytosis  (n=19,  6.25%) | P-value |
| --- | --- | --- | --- | --- | --- |
| Age, years | 57.6 ± 12.2 | 55.9 ± 13.3 | 57.5 ± 14.4 | 57.3 ± 14.7 | 0.79 |
| Sex |  |  |  |  | <0.01 |
| Female | 111 (79.9) | 62 (63.9) | 23 (46.9) | 9 (47.4) |  |
| Male | 28 (20.1) | 35 (36.1) | 26 (53.1) | 10 (52.6) |  |
| Premorbid mRS |  |  |  |  | 0.80 |
| 0 | 135 (97.1) | 93 (95.9) | 47 (95.9) | 19 (100.0) |  |
| 1 | 3 (2.2) | 2 (2.1) | 2 (4.1) | 0 (0.0) |  |
| 2 | 1 (0.7) | 2 (2.1) | 0 (0.0) | 0 (0.0) |  |
| History of hypertension | 59 (42.4) | 39 (40.2) | 21 (42.9) | 9 (47.4) | 0.95 |
| History of diabetes | 8 (5.8) | 6 (6.2) | 9 (18.4) | 4 (21.1) | 0.01 |
| Hunt & Hess classification |  |  |  |  | 0.02 |
| 1 | 29 (20.9) | 14 (14.4) | 4 (8.2) | 3 (15.8) |  |
| 2 | 42 (30.2) | 27 (27.8) | 13 (26.5) | 3 (15.8) |  |
| 3 | 36 (25.9) | 28 (28.9) | 18 (36.7) | 3 (15.8) |  |
| 4 | 26 (18.7) | 18 (18.6) | 11 (22.4) | 4 (21.1) |  |
| 5 | 6 (4.3) | 10 (10.3) | 3 (6.1) | 6 (31.6) |  |
| WFNS grade |  |  |  |  | 0.01 |
| 1 | 71 (51.1) | 37 (38.1) | 17 (34.7) | 6 (31.6) |  |
| 2 | 24 (17.3) | 16 (16.5) | 12 (24.5) | 1 (5.3) |  |
| 3 | 4 (2.9) | 5 (5.2) | 4 (8.2) | 2 (10.5) |  |
| 4 | 30 (21.6) | 23 (23.7) | 9 (18.4) | 2 (10.5) |  |
| 5 | 10 (7.2) | 16 (16.5) | 7 (14.3) | 8 (42.1) |  |
| Modified Fisher grade |  |  |  |  | 0.05 |
| 1 | 36 (25.9) | 14 (14.4) | 7 (14.3) | 3 (15.8) |  |
| 2 | 9 (6.5) | 10 (10.3) | 0 (0.0) | 0 (0.0) |  |
| 3 | 52 (37.4) | 33 (34.0) | 23 (46.9) | 6 (31.6) |  |
| 4 | 42 (30.2) | 40 (41.2) | 19 (38.8) | 10 (52.6) |  |
| Intracerebral hemorrhage | 23 (16.5) | 19 (19.6) | 14 (28.6) | 7 (36.8) | 0.09 |
| Global cerebral edema | 48 (34.8) | 38 (39.6) | 14 (28.6) | 8 (42.1) | 0.55 |
| Subarachnoid hemorrhage  early brain edema score |  |  |  |  | 0.41 |
| 0 | 9 (6.5) | 8 (8.3) | 0 (0.0) | 1 (5.3) |  |
| 1 | 7 (5.1) | 6 (6.2) | 3 (6.1) | 2 (10.5) |  |
| 2 | 53 (38.4) | 28 (29.2) | 19 (38.8) | 4 (21.1) |  |
| 3 | 23 (16.7) | 16 (16.7) | 13 (26.5) | 6 (31.6) |  |
| 4 | 46 (33.3) | 38 (39.6) | 14 (28.6) | 6 (31.6) |  |
| Aneurysm location |  |  |  |  | 0.73 |
| ACA | 6 (4.3) | 4 (4.1) | 2 (4.1) | 2 (10.5) |  |
| ACoA | 48 (34.5) | 37 (38.1) | 15 (30.6) | 3 (15.8) |  |
| ICA | 14 (10.1) | 7 (7.2) | 6 (12.2) | 2 (10.5) |  |
| MCA | 24 (17.3) | 24 (24.7) | 14 (28.6) | 7 (36.8) |  |
| PCoA | 33 (23.7) | 16 (16.5) | 8 (16.3) | 4 (21.1) |  |
| PCA | 3 (2.2) | 0 (0.0) | 0 (0.0) | 0 (0.0) |  |
| VA/Cerebellar | 7 (5.0) | 7 (7.2) | 2 (4.1) | 1 (5.3) |  |
| BA | 4 (2.9) | 2 (2.1) | 2 (4.1) | 0 (0.0) |  |
| Aneurysm circulation |  |  |  |  | 0.91 |
| Anterior | 125 (89.9) | 88 (90.7) | 45 (91.8) | 18 (94.7) |  |
| Posterior | 14 (10.1) | 9 (9.3) | 4 (8.2) | 1 (5.3) |  |
| Onset to arrival time, h | 1.5 [0.7-5.0] | 1.3 [0.7-4.1] | 1.9 [1.0-3.7] | 1.5 [0.5-2.1] | 0.38 |
| Onset to treatment time, h | 7.4 [4.8-11.8] | 7.0 [5.3-11.3] | 6.5 [4.8-11.8] | 6.2 [4.6-8.9] | 0.87 |
| Aneurysm treatment modality |  |  |  |  | <0.01 |
| Surgical | 41 (29.5) | 58 (59.8) | 26 (53.1) | 11 ( 57.9) |  |
| Endovascular | 98 (70.5) | 39 (40.2) | 23 (46.9) | 8 (42.1) |  |
| External ventricular drainage | 34 (24.5) | 36 (37.1) | 20 (40.8) | 10 (52.6) | 0.02 |
| Lumbar drainage | 70 (50.4) | 44 (45.4) | 18 (36.7) | 10 (52.6) | 0.39 |
| TCD vasospasm | 66 (51.2) | 53 (58.2) | 38 (80.9) | 8 (72.7) | <0.01 |
| Angiographic vasospasm | 33 (24.8) | 36 (39.1) | 27 (55.1) | 4 (30.8) | <0.01 |
| Intra-arterial spasmolysis | 20 (14.4) | 20 (20.6) | 22 (44.9) | 3 (15.8) | <0.01 |
| Secondary infarction | 24 (17.3) | 18 (18.6) | 6 (12.2) | 8 (42.1) | 0.04 |
| Shunt dependency | 16 (11.5) | 16 (16.5) | 15 (30.6) | 11 (57.9) | <0.01 |
| mRS at 6 month |  |  |  |  | <0.01 |
| 0-2 | 105 (75.5) | 60(61.9) | 28 (57.1) | 8 (42.1) |  |
| 3-6 | 34 (24.5) | 36 (37.1) | 21 (42.9) | 11 (57.9) |  |

Values are n (%), mean ± standard deviation, or median [interquartile range]; mRS = modified Rankin Score; WFNS = World Federal Neurosurgical Society; SEBES= Subarachnoid hemorrhage early brain edema score; ACA = anterior cerebral artery ; AcoA = anterior communicating artery; ICA = internal carotid artery ; MCA = middle cerebral artery; PCoA = posterior communicating artery; PCA = posterior cerebral artery; BA = Basilar artery; VA = Vertebral artery; TCD = transcranial *Doppler*

**Supplemental Table 2. Comparison of patient characteristics and outcomes across lymphocyte cluster**

|  | No  lymphopenia  (n=54,  17.8%) | Borderline  normal  (n=100,  32.9%) | Early transient lymphopenia  (n=61,  20.1%) | Early prolonged  lymphopenia  (n=89,  29.3%) | P-value |
| --- | --- | --- | --- | --- | --- |
| Age, year | 53.8 ± 13.9 | 58.1 ± 11.7 | 54.5 ± 13.0 | 59.5 ± 13.5 | 0.02 |
| Sex |  |  |  |  | 0.32 |
| Female | 31 (57.4) | 72 (72.0) | 41 (67.2) | 61 (68.5) |  |
| Male | 23 (42.6) | 28 (28.0) | 20 (32.8) | 28 (31.5) |  |
| Premorbid mRS |  |  |  |  | 0.73 |
| 0 | 53 (98.1) | 95 (95.0) | 60 (98.4) | 86 (96.6) |  |
| 1 | 1 (1.9) | 4 (4.0) | 0 (0.0) | 2 (2.2) |  |
| 2 | 0 (0.0) | 1 (1.0) | 1 (1.6) | 1 (1.1) |  |
| History of hypertension | 25 (46.3) | 35 (35.0) | 22 (36.1) | 46 (51.7) | 0.08 |
| History of diabetes | 6 (11.1) | 6 (6.0) | 5 (8.2) | 10 (11.2) | 0.57 |
| Hunt & Hess Classification |  |  |  |  | 0.01 |
| 1 | 10 (18.5) | 18 (18.0) | 8 (13.1) | 14 (15.7) |  |
| 2 | 21 (38.9) | 31 (31.0) | 12 (19.7) | 21 (23.6) |  |
| 3 | 19 (35.2) | 24 (24.0) | 25 (41.0) | 17 (19.1) |  |
| 4 | 3 (5.6) | 20 (20.0) | 10 (16.4) | 26 (29.2) |  |
| 5 | 1 (1.9) | 7 (7.0) | 6 (9.8) | 11 (12.4) |  |
| WFNS grade |  |  |  |  | 0.01 |
| 1 | 32 (59.3) | 49 (49.0) | 20 (32.8) | 30 (33.7) |  |
| 2 | 13 (24.1) | 16 (16.0) | 12 (19.7) | 12 (13.5) |  |
| 3 | 3 (5.6) | 3 (3.0) | 3 (4.9) | 6 (6.7) |  |
| 4 | 2 (3.7) | 22 (22.0) | 15 (24.6) | 25 (28.1) |  |
| 5 | 4 (7.4) | 10 (10.0) | 11 (18.0) | 16 (18.0) |  |
| Modified Fisher grade |  |  |  |  | 0.77 |
| 1 | 14 (25.9) | 22 ( 22.0) | 9 (14.8) | 15 (16.9) |  |
| 2 | 3 (5.6) | 8 (8.0) | 5 (8.2) | 3 (3.4) |  |
| 3 | 20 (37.0) | 35 (35.0) | 24 (39.3) | 35 (39.3) |  |
| 4 | 17 (31.5) | 35 (35.0) | 23 (37.7) | 36 (40.4) |  |
| Intracerebral hemorrhage | 8 (14.8) | 13 (13.0) | 18 (29.5) | 24 (27.0) | 0.02 |
| Global cerebral edema | 14 (25.9) | 32 (32.0) | 29 (48.3) | 33 (37.5) | 0.07 |
| Subarachnoid hemorrhage  early brain edema score |  |  |  |  | 0.06 |
| 0 | 2 (3.7) | 9 (9.0) | 2 (3.3) | 5 (5.7) |  |
| 1 | 4 (7.4) | 7 (7.0) | 1 (1.7) | 6 (6.8) |  |
| 2 | 23 (42.6) | 39 (39.0) | 12 (20.0) | 30 (34.1) |  |
| 3 | 12 (22.2) | 13 (13.0) | 18 (30.0) | 15 (17.0) |  |
| 4 | 13 (24.1) | 32 (32.0) | 27 (45.0) | 32 (36.4) |  |
| Aneurysm location |  |  |  |  | 0.11 |
| ACA | 4 (7.4) | 2 (2.0) | 3 (4.9) | 5 (5.6) |  |
| ACoA | 19 (35.2) | 39 ( 39.0) | 23 (37.7) | 22 (24.7) |  |
| ICA | 3 (5.6) | 9 (9.0) | 4 (6.6) | 13 (14.6) |  |
| MCA | 15 (27.8) | 12 (12.0) | 19 (31.1) | 23 (25.8) |  |
| PCoA | 9 (16.7) | 27 (27.0) | 8 (13.1) | 17 (19.1) |  |
| PCA | 0 (0.0) | 2 (2.0) | 0 (0.0) | 1 (1.1) |  |
| VA/Cerebellar | 1 (1.9) | 6 (6.0) | 3 (4.9) | 7 (7.9) |  |
| BA | 3 (5.6) | 3 (3.0) | 1 (1.6) | 1 (1.1) |  |
| Aneurysm circulation |  |  |  |  | 0.76 |
| Anterior | 50 (92.6) | 89 (89.0) | 57 (93.4) | 80 (89.9) |  |
| Posterior | 4 (7.4) | 11 (11.0) | 4 (6.6) | 9 (10.1) |  |
| Onset to arrival time, h | 2.2 [1.0-5.4] | 1.6 [0.8 - 4.1] | 1.3 [0.6-3.9] | 1.4 [0.7-4.0] | 0.39 |
| Onset to treatment time, h | 7.0 [5.0-13.4] | 7.4 [4.8-11.0] | 7.0 [4.8-10.8] | 7.2 [5.0-12.0] | 0.97 |
| Aneurysm treatment modality |  |  |  |  | <0.01 |
| Surgical | 15 (27.8) | 31 (31.0) | 39 (63.9) | 51 (57.3) |  |
| Endovascular | 39 (72.2) | 69 (69.0) | 22 (36.1) | 38 (42.7) |  |
| External ventricular drainage | 14 (25.9) | 30 (30.0) | 24 (39.3) | 32 (36.0) | 0.38 |
| Lumbar drainage | 26 (48.1) | 49 (49.0) | 27 (44.3) | 40 (44.9) | 0.92 |
| TCD vasospasm | 26 (54.2) | 53 (55.8) | 39 (72.2) | 47 (58.0) | 0.19 |
| Angiographic vasospasm | 14 (27.5) | 30 ( 30.9) | 28 (49.1) | 28 (34.1) | 0.07 |
| Intra-arterial spasmolysis | 11 (20.4) | 18 (18.0) | 17 (27.9) | 19 (21.3) | 0.52 |
| Secondary infarction | 7 (13.0) | 12 (12.0) | 14 (23.0) | 23 (25.8) | 0.05 |
| Shunt dependency | 6 (11.1) | 13 (13.0) | 17 (27.9) | 22 (24.7) | 0.02 |
| mRS at 6 month |  |  |  |  | 0.01 |
| 0-2 | 46 (85.2) | 75 (75.0) | 35 (57.4) | 45 (50.6) |  |
| 3-6 | 8 (14.8) | 25 (25.0) | 26 (42.6) | 43 (48.3) |  |

Values are n (%), mean ± standard deviation, or median [interquartile range]; mRS = modified Rankin Score; WFNS = World Federal Neurosurgical Society; SEBES= Subarachnoid hemorrhage early brain edema score; ACA = anterior cerebral artery; AcoA = anterior communicating artery; ICA = internal carotid artery ; MCA = middle cerebral artery; PCoA = posterior communicating artery; PCA = posterior cerebral artery; BA = Basilar artery; VA = Vertebral artery; TCD = transcranial *Doppler*

**Supplemental Table 3. Multivariable analysis of association between immune cell clusters and clinical outcomes**

|  | TCD vasospasm | Angiographic vasospasm | Intra-arterial spasmolysis | Secondary infarction | Shunt dependency | mRS 3-6 at 6 month |
| --- | --- | --- | --- | --- | --- | --- |
| No neutrophilia | Referent | Referent | Referent | Referent | Referent | Referent |
| Resolving neutrophilia | 1.06 [0.52-2.13]  (p=0.88) | 2.25 [1.06-4.76]  (p=0.03) | 1.52 [0.62-3.72]  (p=0.36) | 1.24 [0.51-3.05]  (p=0.63) | 1.36 [0.40-4.63]  (p=0.62) | 3.02 [1.19-7.71]  (p=0.02) |
| Progressive neutrophilia | 2.16 [0.83-5.57]  (p=0.11) | 4.11 [1.60-10.55]  (p<0.01) | 6.53 [2.30-18.51]  (p<0.01) | 1.66 [0.59-4.64]  (p=0.34) | 5.05 [1.51-16.84]  (p=0.01) | 8.14 [2.84-23.33]  (p<0.01) |
| Persistent neutrophilia | 4.58 [1.41-14.81]  (p=0.01) | 4.88 [1.76-13.52]  (p<0.01) | 5.62 [1.94-17.30]  (p<0.01) | 1.68 [0.58-4.86]  (p=0.34) | 9.89 [2.96-33.07]  (p<0.01) | 7.24 [2.42-21.65]  (p<0.01) |
| No monocytosis | Referent | Referent | Referent | Referent | Referent | Referent |
| Borderline monocytosis | 0.8 [0.41-1.56]  (p=0.51) | 1.64 [0.82-3.29]  (p=0.16) | 1.54 [0.71-3.35]  (p=0.28) | 0.83 [0.39-1.79]  (p=0.63) | 0.96 [0.38-2.44]  (p=0.93) | 1.42 [0.68-2.97]  (p=0.35) |
| Early  monocytosis | 2.97 [1.14-7.75]  (p=0.03) | 3.48 [1.50-8.07]  (p<0.01) | 5.13 [2.09-12.62]  (p<0.01) | 0.46 [0.16-1.34]  (p=0.16) | 2.61 [0.96-7.10]  (p=0.06) | 1.64 [0.66-4.09]  (p=0.29) |
| Delayed monocytosis | 2.07 [0.42-10.15]  (p=0.37) | 0.89 [0.20-4.08]  (p=0.88) | 1.28 [0.28-5.80]  (p=0.75) | 2.86 [0.85-9.65]  (p=0.09) | 7.94 [2.08-30.29]  (p<0.01) | 1.61[0.44-5.87]  (p=0.47) |
| No lymphopenia | Referent | Referent | Referent | Referent | Referent | Referent |
| Borderline normal lymphocyte counts | 1.46 [0.63-3.37]  (p=0.38) | 1.39 [0.57-3.37]  (p=0.47) | 1.08 [0.42-2.77]  (p=0.88) | 0.80 [0.27-2.40]  (p=0.69) | 0.88 [0.27-2.85]  (p=0.83) | 1.49 [0.51-4.32]  (p=0.47) |
| Early transient lymphopenia | 1.11 [0.45-2.77]  (p=0.82) | 1.98 [0.76-5.18]  (p=0.16) | 1.08 [0.40-2.94]  (p=0.88) | 1.30 [0.43-3.91]  (p=0.64) | 2.05 [0.62-6.78]  (p=0.24) | 3.39 [1.21-10.22]  (p=0.03) |
| Early prolonged lymphopenia | 1.79 [0.67-4.82]  (p=0.25) | 1.34 [0.52-3.49]  (p=0.54) | 1.08 [0.40-2.89]  (p=0.88) | 1.68 [0.59-4.73]  (p=0.33) | 1.63 [0.51-5.26]  (p=0.41) | 3.43 [1.20-9.79]  (p=0.02) |

Values are adjusted odds ration [95% confidence interval] (p-value). TCD = transcranial *Doppler*; mRS = modified Rankin score
